# Glucose-Derived Raspberry Ketone Produced via Engineered *Escherichia coli* Metabolism

**DOI:** 10.3389/fbioe.2022.843843

**Published:** 2022-02-14

**Authors:** Shunsuke Masuo, Chisa Saga, Kurumi Usui, Yuma Sasakura, Yukie Kawasaki, Naoki Takaya

**Affiliations:** Faculty of Life and Environmental Sciences, Microbiology Research Center for Sustainability, University of Tsukuba, Tsukuba, Japan

**Keywords:** raspberry ketone, flavor agent, microbial production, plant secondary metabolite, metabolic engieering

## Abstract

The demand for raspberry ketone (RK) as a plant-based natural flavoring agent is high, but natural RK is one of the most expensive flavor compounds due to its limited content in plants. Here, we produced RK *de novo* from simple carbon sources in *Escherichia coli.* We genetically engineered *E. coli* metabolism to overproduce the metabolic precursors tyrosine and *p*-coumaric acid and increase RK production. The engineered *E. coli* produced 19.3- and 1.9 g/L of tyrosine and *p*-coumaric acid from glucose, respectively. The *p*-coumaric acid CoA ligase from *Agrobacterium tumefaciens* and amino acid substituted benzalacetone synthase of *Rhemu palmatum* (Chinese rhubarb) were overexpressed in *E. coli* overproducing *p*-coumaric acid*.* The overexpression of *fabF*, encoding β-ketoacyl-acyl carrier protein synthetase II increased intracellular malonyl-CoA, the precursor of benzalacetone synthase for RK biosynthesis, and improved RK production. Fed-batch cultures given glucose as a carbon source produced 62 mg/L of RK under optimized conditions. Our production system is inexpensive and does not rely on plant extraction; thus, it should significantly contribute to the flavor and fragrance industries.

## 1 Introduction

The raspberry phenylbutanoid ketone 4-(4-hydroxyphenyl)butan-2-one (RK) is a natural flavor in plants such as raspberries, grapes, peaches, and rhubarb. The berry flavor of RK with a low odor threshold is used as a food additive to create various aromas such as cherry, strawberry, kiwi and other fruits (Beekwilder et al., 2007; Bredsdorff et al., 2015; Sun et al., 2021). The alleged health benefits of RK include weight reduction (Morimoto et al., 2005; Wang et al., 2012) and skin lightening (Harada et al., 2008) and it is in high demand in the supplements and cosmetics industries (Milke et al., 2020; Vandamme and Soetaert, 2002). As the demand for RK is second only to vanillin, the potential of RK in the natural flavor market RK is 6–10 million Euros (Feron and Wache 2005). However, RK is difficult to cost-effectively produce from plants due to low contents. For example, raspberries contain only 1 to 4 mg/kg of RK (Larsen et al., 1991; Beekwilder et al., 2007), and extraction costs are high (Böker et al., 2001). Thus, the market for naturally occurring RK as a flavoring agent is United States $3,000–$20,000/kg (Milke et al., 2020). Chemically synthesized RK (Malkar and Yadav 2019) is not regarded as a natural flavor by United States and EU regulations, and it is essentially unacceptable to some consumers. However, microbial fermentation is an alternative strategy that allows inexpensive mass production of RK without the need for extraction from plants.

**FIGURE 1:**
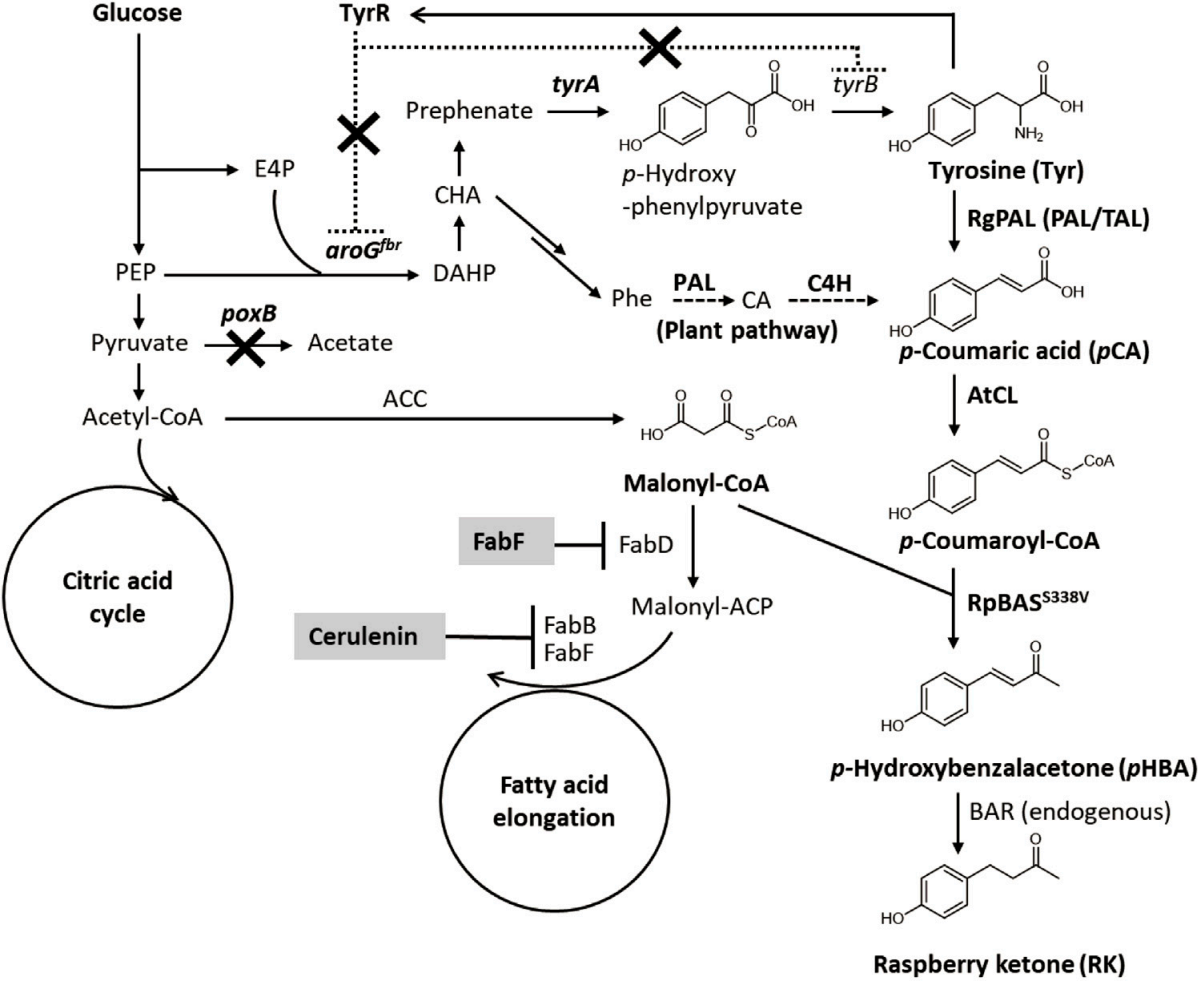
Metabolic network for RK biosynthesis from glucose in engineered *E. coli*. ACC, acetyl-CoA carboxylase; ACP, acyl carrier protein; aroG^fbr^, feedback resistant isozyme of DAHP synthase; BAR, benzalacetone reductase; BAS, benzalacetone synthase; CA, cinnamic acid; CL, 4-coumarate CoA ligase; C4H, cinnamate-4-hydroxylase; DAHP, 3-deoxy-D-arabino-heptulosonate 7-phosphate; E4P, erythrose 4-phosphate; FabB, β-ketoacyl-ACP synthase I; FabD, malonyl-CoA-ACP transacylase; FabF, β-ketoacyl-ACP synthase II; PAL, phenylalanine ammonia lyase; PEP, phosphoenolpyruvate; Phe, phenylalanine; *poxB*, pyruvate oxidase; TAL, tyrosine ammonia lyase; *tyrA*, chorismate mutase/prephenate dehydrogenase; *tyrB*, tyrosine aminotransferase; TyrR, transcriptional repressor of aromatic amino acid biosynthesis genes.

The RK biosynthesis pathways and mechanisms in raspberries and rhubarb have been investigated (Borejsza-Wysocki and Hrazdina 1994; Abe et al., 2001). The pathway starts from *p*-coumaroyl-CoA (Figure 1), which is a ubiquitous intermediate of the plant lignin biosynthetic pathway. During RK synthesis, the non-oxidative deamination of phenylalanine is catalyzed by phenylalanine ammonia lyase (PAL) and followed by hydroxylation and CoA-activation catalyzed by cinnamate-4-hydroxylase (C4H) and *p*-coumarate CoA ligase (CL) to produce *p*-coumaroyl-CoA. Thereafter, *p*-coumaroyl-CoA is condensed with malonyl-CoA to generate *p*-hydroxybenzalacetone by the activity of benzalacetone synthase (BAS), which is a type III polyketide synthase. The resulting *p*-hydroxybenzalacetone is reduced by the NADPH-dependent benzalacetone reductase (BAR) to yield RK (Figure 1).

Microbial RK has been produced using genetically manipulated microorganisms such as yeast, *Escherichia coli* and other bacteria (Lee at al. 2016; Wang et al., 2019; Milke et al., 2020). The common approach converts *p*-coumaric acid as a starting material to RK in host cells producing heterogenous CL, BAS and BAR that originate from plants. Host *E. coli* and *Corynebacterium glutamicum* cells convert *p*-coumaric acid to RK with titers of 91.0 and 99.8 mg/l, respectively (Wang et al., 2019; Milke et al., 2020). However, few efforts have been made to produce RK *de novo* using renewable carbon sources. One exception is a wine yeast that generates plant PAL, C4H, and the synthetic CL and BAS fusion enzyme, and this yeast produces 3.5 mg/l of RK in grape juice medium (Lee at al. 2016), which is far lower than that yielded using *p*-coumaric acid as the raw material. The low titer could be due to the low availability of *p*-coumaroyl-CoA and malonyl-CoA that are substrates of BAS, the key enzyme of RK biosynthesis. Here, we showed that improving *p*-coumaric acid and malonyl-CoA availability significantly increased the productivity of microbial *de novo* RK synthesis. We constructed *E. coli* that generated abundant *p*-coumaric acid and malonyl-CoA by metabolic engineering and chemical stimulation. After gene selection and stepwise culture optimization, our recombinant *E. coli* produced RK biosynthesis enzymes and fermented glucose to produce 62 mg/L of RK.

## 2 Materials and Methods

### 2.1 Strains, Materials, and Instrumentation

Supplementary Table S1 lists the strains used in this study. We produced RK and constructed plasmids using *E. coli* BL21 (DE3) and *E. coli* JM109 (Novagen, Madison, WI, United States), respectively. We purchased 4-coumaroyl-CoA from Sigma–Aldrich (St. Louis, MO, United States) and l-tyrosine, *p*-coumaric acid, and *p*-hydroxybenzalacetone from Wako Chemicals (Tokyo, Japan). Plasmids were constructed using KOD One PCR Master Mix (Toyobo, Osaka, Japan), restriction enzymes (Takara Bio Inc., Shiga, Japan), Ligation high Ver. 2 (Toyobo) and NEBuilder HiFi DNA Assembly Master Mix (New England Biolabs Inc., Ipswich, MA, United States). Metabolites were analyzed by high-performance liquid chromatography (HPLC) using a 1200 infinity photodiode array detector (Agilent Technologies Inc., Santa Clara, CA, United States), and by LC-ESI-MS/MS and GC-MS using LCMS-8045 and QP-2010 mass spectrometers (both from Shimadzu, Kyoto, Japan).

### 2.2 Plasmid Construction and *E. coli* Gene Knockout

Supplementary Table S2 lists the plasmids used in this study. Nucleotide fragments of the *tyrA* gene were amplified by PCR using *E. coli* MG1655 genomic DNA and primers (Supplementary Table S3), digested with NcoI and BamHI, and cloned into pETduet-1 (Novagen) that was also digested with these enzymes to generate pET-tyrA. Plasmid pET-FevV for producing TAL (*fevV* from *Streptomyces* sp. WK-5344), was provided by Dr. Kawaguchi (Kawaguchi et al., 2017). Nucleotide sequences of the PAL genes of *Camellia sinensis* (Matsumoto et al., 1994) and *Lithospermum erythrorhizon* (Yazaki et al., 1995) were optimized according to *E. coli* codon usage (accession numbers; MZ439822 and MZ439823, Cspal and Lepal), synthesized and cloned into pUC57 (Genscript Biotech Corp., Piscataway, NJ, United States) to generate pUC-Cspal and pUC-Lepal. These plasmids were digested with NdeI and EcoRI and the resulting PAL gene fragments were cloned into pET-28b (Novagen) to obtain pET-Cspal and pET-Lepal, respectively. Plasmid pET28a-*pal* (Zhu et al., 2013) was digested with NdeI and XhoI to obtain *Rhodotorula glutinis* PAL gene fragments*,* and these were cloned into pRSFduet-1 (Novagen) to generate pRSF-Rgpal. The *Agrobacterium tumefaciens* 4-coumarate CoA ligase gene (Atu1416) was amplified by PCR using *A. tumefaciens* C58 genomic DNA and primers (Supplementary Table S3), digested with EcoRI and SalI, and cloned into pCDFduet-1 (Novagen) to generate pCDF-AtCL. The *Rubus idaeus* and *Rhemu palmatum* BAS genes were codon optimized (accession numbers; MZ439820 and MZ439821), synthesized, and cloned into pEX-A2 to obtain pEX-RiBAS and pEX-RpBAS (Eurofins Genomics Inc., Tokyo, Japan), respectively. We mutated BAS genes using QuickChange Site-directed Mutagenesis Kits (Agilent Technologies) and primers (Supplementary Table S3) to generate pEX-RiBAS^S338V^ pEX-RpBAS^S331V^. The DNA fragments of RiBAS and RpBAS genes were digested with NdeI and XhoI and cloned into pCDF-AtCL to generate pCDF-AtCL-RiBAS, pCDF-AtuCL-RpBAS, pCDF-AtCL-RiBAS^S338V^ and pCDF-AtuCL-RpBAS^S331V^, respectively. We amplified *fabF* by PCR using *E. coli* MG1655 genomic DNA and primers (Supplementary Table S3), then assembled it with pET28b that was digested with NcoI and XhoI to generate pET-fabF. Fragments of DNA containing the T7lac promoter, *fabF*, and T7 terminator sequences were amplified by PCR using pET-fabF and primers (Supplementary Table S3), then assembled with pCDF-AtCL-RpBAS that was digested with PacI to generate pCDF-AtCL-RpBAS-fabF. The *tyrR* and *poxB* genes of BL21 (DE3) were disrupted using the Red/ET recombination system (Gene Bridges, Heidelberg, Germany) with the described primers (Supplementary Table S3) to generate ΔtyrR and ΔpoxB strains (Masuo et al., 2016).

### 2.3 Fermentation

Recombinant *E. coli* BL21 (DE3) strains were cultured in 3 ml of LB medium, then 2 ml portions were inoculated into 500-ml conical flasks containing 100 ml of fermentation medium (10 g glucose, 10 g tryptone, 5 g yeast extract, 24 g Na_2_HPO_4_, 12 g KH_2_PO_4_, 0.5 g NaCl, 1 g NH_4_Cl, 0.5 g MgSO_4_ 7H_2_O, 15 mg CaCl_2_, 50 mg thiamine-HCl and 2 ml of trace element solution/L; Fujita et al., 2013) or modified fermentation medium (10 g glucose, 10 g tryptone, 5 g yeast extract, 12 g Na_2_HPO_4_, 6 g KH_2_PO_4_, 42 g MOPS, 0.5 g NaCl, 10 g (NH4)2SO4, 0.5 g MgSO_4_ 7H_2_O, 15 mg CaCl_2_, 50 mg thiamine-HCl and 2 ml of trace element solution/L). The flasks were rotary-shaken at 120 rpm and 30°C for 3 h under aerobic conditions unless otherwise stated. When the OD_600_ reached 0.6, 0.5 mM isopropyl-β-D-thiogalactoside (IPTG) was added, then the cells were further incubated for the indicated amounts of time. Fed-batch cultures in a 1.0-L BMJ-01 fermenter (Biott, Tokyo, Japan) containing 0.5 L of fermentation or modified fermentation medium were agitated at 550 rpm, 30°C, and aerated at 1.0 L/min. When the OD_600_ reached 0.6, 0.1 mM IPTG was added. Peristaltic pumps fed the cultures with 500 g/L of glucose when the glucose concentration dipped below 1.5 g/L. The pH was monitored using an electrode and maintained between 7.0 and 7.1 by adding 10% NH_4_OH.

### 2.4 Bioconversion

*Escherichia coli* BL21 (DE3) harboring either pET-FevV, pET-28a-*pal*, pET-Cspal or pET-Lepal was cultured in 3 ml of LB medium, then 2 ml portions were inoculated into 100 ml of LB medium and rotary-shaken at 120 rpm at 30°C under aerobic conditions. When the OD_600_ of the cultures reached 0.6, the cultures were incubated for 18 h with 0.5 mM IPTG, then the cells were sedimented by centrifugation at 3,000 × g for 10 min. The cells were washed with 50 mM Tris-HCl (pH 7.0), suspended in 50 mM Tris-HCl (pH 8.0) containing 1.8 g/L of tyrosine and incubated at 30°C with agitation at 120 rpm.

### 2.5 GC-MS Analysis

Culture supernatants were acidified with formic acid and extracted twice with equal amounts ethyl acetate. The ethyl acetate was evaporated, then the extracts were dissolved in methanol and analyzed by GC-MS equipped with a ZB-5MS capillary column (30 m × 0.32 mm internal diameter × 0.25-μm film thickness: Phenomenex, Torrance, CA, United States). The injection and ion-source temperatures were 250 and 200°C, respectively. The linear velocity of the carrier helium gas was 45 cm/s. The oven temperature was controlled at 40°C for 4 min, increased to 250°C at a rate of 12°C/min, then maintained at 250°C for 7 min.

### 2.6 Determination of Metabolite Concentrations

Tyrosine and *p*-coumaric acid were quantified using HPLC and a TSKgel® ODS-100V column (4.6 mm × 25 cm, particle size 3 μm, Tosoh, Tokyo, Japan). The initial mobile phase comprised 95%:5% 10 mM ammonium formate (pH 7.0): acetonitrile for 8 min, followed by an increase to 50% acetonitrile for 6 min and maintained for 2 min. The flow rate of 0.8 ml min^−1^ and the column temperature of 30°C were maintained throughout the analysis.

We quantified RK and intermediates using the multiple-reaction monitoring mode on the LC-ESI-MS/MS under the following conditions: capillary voltage, 4.5 kV; desolvation line, 250°C; heat block, 400°C; nebulizer nitrogen gas 3 L/min; drying gas, 10 L/min. Compounds of interest were separated by the LC system equipped with a 150 × 2.1 mm ACQUITY UPLC CSH™ C_18_ 2.1 × 150 mm (Waters, Corp., Milford, MA United States) with particle and pore sizes of 1.7 μm and 130 Å , respectively. The initial mobile phase was solvent A (0.025% formic acid) for 4 min. The concentration of solvent B (acetonitrile) was increased to 50% for 11 min, then maintained for 1 min. The column was re-equilibrated for 4 min. Malonyl-CoA was separated using 5 mM ammonium formate (pH 7.0) containing 50% acetonitrile as the mobile phase in isocratic mode. The flow rate of 0.4 ml min^−1^ and the column temperature at 40°C were maintained throughout the analysis. The MRM-transitions were *m*/*z* 180.05 to 163.15 (for tyrosine, negative ion mode), *m*/*z* 163.05 to 119.05 (*p*-coumaric acid, negative ion mode), *m*/*z* 165.05 to 147.10 (p-hydroxybenzalacetone, positive ion mode), *m*/*z* 854.05 to 303.00 (malonyl-CoA, positive ion mode) and *m*/*z* 165.10 to 107.10 (RK, positive ion mode). The dwell time, Q1 pre-bias, collision energy and Q3 pre-bias were set at 100 ms, 20 V, 14 eV, 17 V for tyrosine, 100 ms, 17 V, 14 eV, 21 V for *p*-coumaric acid, 100 ms, −12vV, −12 eV, −15 V for *p*-hydroxybenzalacetone, 100 ms, −34 V, −41 eV, −30 V for malonyl-CoA, and 100 ms, −14 V, −12 eV, −11 V for RK.

## 3 Results

### 3.1 Metabolic Engineering of *E. coli* to Produce *p*-Coumaric Acid

**FIGURE 2:**
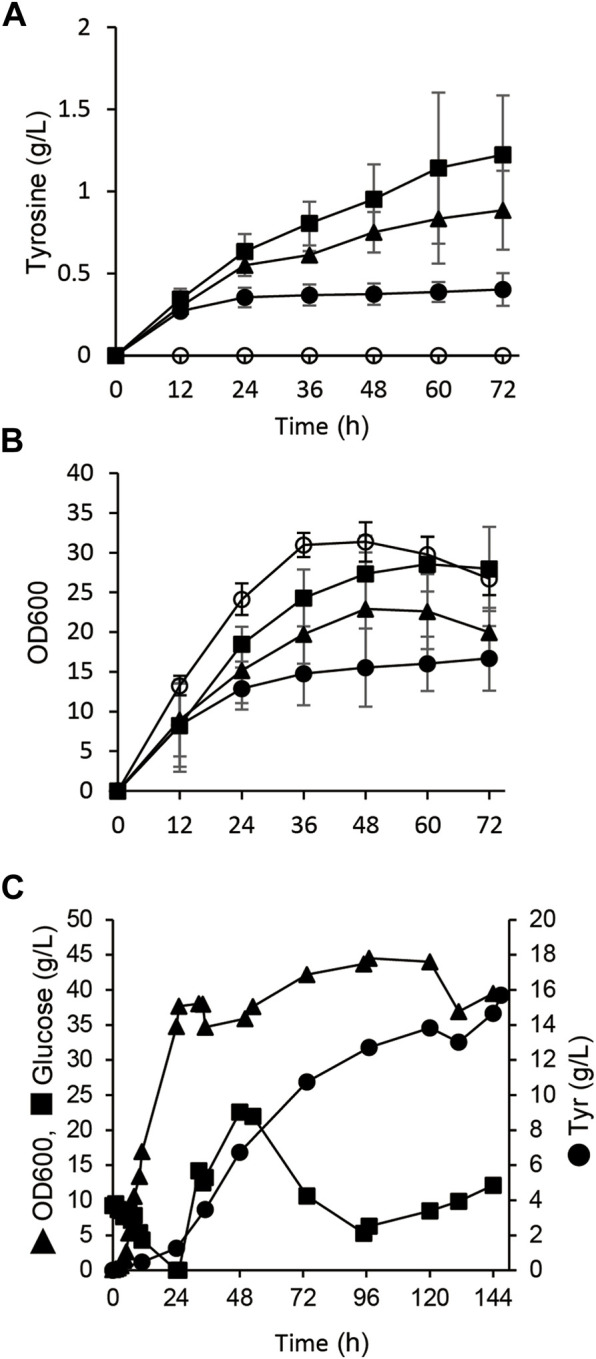
Tyrosine production from glucose by *E. coli* with engineered metabolism. **(A,B)** Time-dependent tyrosine production **(A)** and growth **(B)** of *E. coli* BL21 (DE3) (closed circles), *ΔpoxB* (closed triangles) and *ΔtyrR* (closed squares) harboring pET-tyrA and pACYC-aroG^fbr^ in fermentation medium at 30°C. *E. coli* BL21 (DE3) harboring pET and pACYC empty plasmids was analyzed as a control (open squares). **(C)** Fed-batch cultures of *ΔtyrR* harboring pET-tyrA and pACYC-aroG^fbr^ in jar fermenters containing 0.5 L of fermentation medium at 30°C and pH 7.1 (maintained by 10% ammonium) were incubated for 24 h, then fed with glucose (0.8 g/L/h). Error bars indicate standard deviation (*n* = 3).

We initially generated *E. coli* that overproduced tyrosine, the precursor of *p*-coumaric acid to construct a microbial *de novo* RK synthesis system (Figure 1). A feedback resistant mutant of 3-deoxy-d-arabinoheptulosonate-7-phosphate (DAHP) synthase (*aroG* ^*fbr*^) and the chorismate mutase/prephenate dehydrogenase gene (*tyrA*) were overexpressed in *E. coli* BL21 (DE3) with the native *aroG* and T7 promoters, respectively (Figure 1). The resulting strain produced 0.4 ± 0.1 g/L of tyrosine in the modified M9 medium, whereas the parental *E. coli* BL21 (DE3) almost did not (Supplementary Figure S1). Another potential parental strain, *E. coli* NST37 (DE3)/*ΔpheLA*, which has an enhanced shikimate pathway (Masuo et al., 2016), produced less tyrosine after the same genetic modification (Supplementary Figure S1B). The *poxB* gene deletion mutant (*ΔpoxB*) harboring *aroG* ^*fbr*^ and *tyrA* expression plasmids produced 0.9 ± 0.2 g/L of tyrosine in the 72-h flask culture (Figure 2A and Table 1). Deletion of *poxB* encoding the pyruvate oxidase that synthesizes acetate from pyruvate (Causey et al., 2004), led to a positive effect on tyrosine production probably due to the altered carbon flux including acetate, phosphoenolpyruvate, and erythrose-4-phosphate generation (Figure 1).

**TABLE 1:** Tyrosine production from glucose by *E. coli* gene deletion mutants.

Strain	Growth rate (h^−1^)	Tyr production (g/L)	Tyr production rate (g/L/h)	Yield (%)
Wild-type (pET-duet, pACYC184)	0.90 ± 0.25	<0.01	<0.00002	n.d.
Wild-type (pET-tyrA, pACYC-aroG4)	0.38 ± 0.08	0.40 ± 0.08	0.007 ± 0.001	1.9
ΔpoxB (pET-tyrA, pACYC-aroG4)	0.52 ± 0.12	0.89 ± 0.08	0.021 ± 0.003	3.8
ΔtyrR (pET-tyrA, pACYC-aroG4)	0.85 ± 0.08	1.2 ± 0.1	0.024 ± 0.002	5.1

Wild-type *E. coli* BL21 (DE3), ΔpoxB and ΔtyrR harboring pET-tyrA and pACYC-aroG4 were cultured in fermentation medium. Growth and tyrosine production rates were calculated from changes in OD_600_ and tyrosine concentrations during culture for 12 and 24 h. The yield was calculated from amounts of tyrosine produced and glucose consumed after 96 h of culture. n.d.: not determined.

A gene deletion mutant of *tyrR* (*ΔtyrR*), which encodes a transcriptional repressor of aromatic amino acid biosynthesis genes (Pittard et al., 2005), harboring the *aroG* ^*fbr*^ and *tyrA* expression plasmids (strain AT1) produced 1.2 ± 0.3 g/L of tyrosine in flask cultures. The production titer of AT1 was 3.0-fold higher than that of parental BL21 (DE3) strain (Figure 2A and Table 1). Growth defect caused by *aroG* ^*fbr*^ and *tyrA* overexpression was restored by gene disruption of *tyrR* (Figures 2A,B). Fed-batch cultures of AT1 with stepwise additions of glucose avoided excessive glucose accumulation in the jar fermenters. The culture generated 15.7 g/L tyrosine with a production yield of 7.4% vs glucose (Figure 2C). Precipitates that appeared on the inner walls of fermentation vessels (Supplementary Figure S2B), contained 1.9 g of tyrosine, and the total amount produced was 19.3 g/L. The culture generated low levels of acetate (<1.0 g L^−1^; Supplementary Figure S2A), indicating decreased metabolic flow of glucose to acetate, which resulted in increased tyrosine production.

**FIGURE 3:**
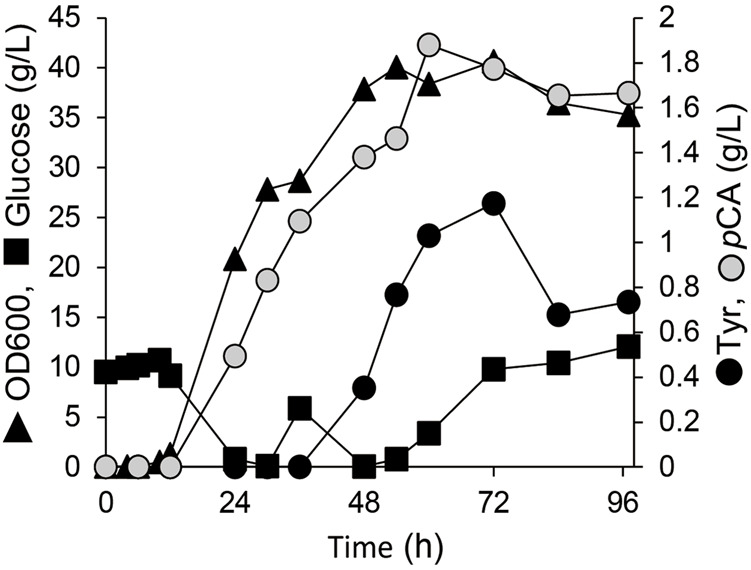
Production of *p*-coumaric acid by *E. coli.* Fed-batch cultures of AT2 strain in jar fermenters containing 0.5 L of fermentation medium at 30°C and pH 7.1 (maintained by 10% ammonium) were incubated for 24 h, then fed with glucose (0.8 g/L/h).

To convert the produced tyrosine to *p*CA, we employed tyrosine ammonia lyase (TAL) or bifunctional PAL that deaminate phenylalanine and tyrosine. We cloned TAL or PAL genes derived from yeast, plant or bacteria into the pRSFduet1 vector under the control of the T7 promoter and the resulting plasmids were introduced into BL21 (DE3). These strains converted tyrosine into *p*CA (Table 2). The *E. coli* producing *Rodotorura glutinis* PAL (RgPAL) had the highest yield, conversion rate and efficiency, and was deemed adequate for *p*CA synthesis *de novo*. We introduced the RgPAL expression plasmid into the AT1 strain and the resulting AT2 strain produced 1.9 g/L of *p*CA from glucose in a fed-batch culture under optimized conditions over a period of 60 h (Figure 3). The culture accumulated >1.0 g/L tyrosine, indicating that PAL reaction limits *p*CA production.

**TABLE 2:** Bioconversion of tyrosine to *p*-coumaric acid by *E. coli* harboring PAL expression plasmids.

PAL/TAL	*p*-Coumaric acid produced (g/L)	*p*-Coumaric acid production rate (g/L/h)	Conversion efficiency (%)
FevV	1.1 ± 0.1	0.095 ± 0.008	66
RgPAL	1.6 ± 0.2	0.80 ± 0.04	89
CsPAL	0.27 ± 0.01	0.05 ± 0.01	17
LePAL	1.5 ± 0.1	0.50 ± 0.06	88

*Escherichia coli* BL21 (DE3) cells (10 mg wet weight), harboring pET-FevV (Kawaguchi et al., 2017), pET28a-*pal* (Zhu et al., 2013), pET-Cspal or pET-Lepal, were each incubated in 1 ml of 100 mM Tris-HCl (pH 8.5) containing 1.8 g/l of tyrosine. Production rates were calculated from the amount of p-coumaric acid produced during the first 0.5 h of incubation.

### 3.2 RK Production by *p*-Coumaric Acid Producing *E. coli*.

**FIGURE 4:**
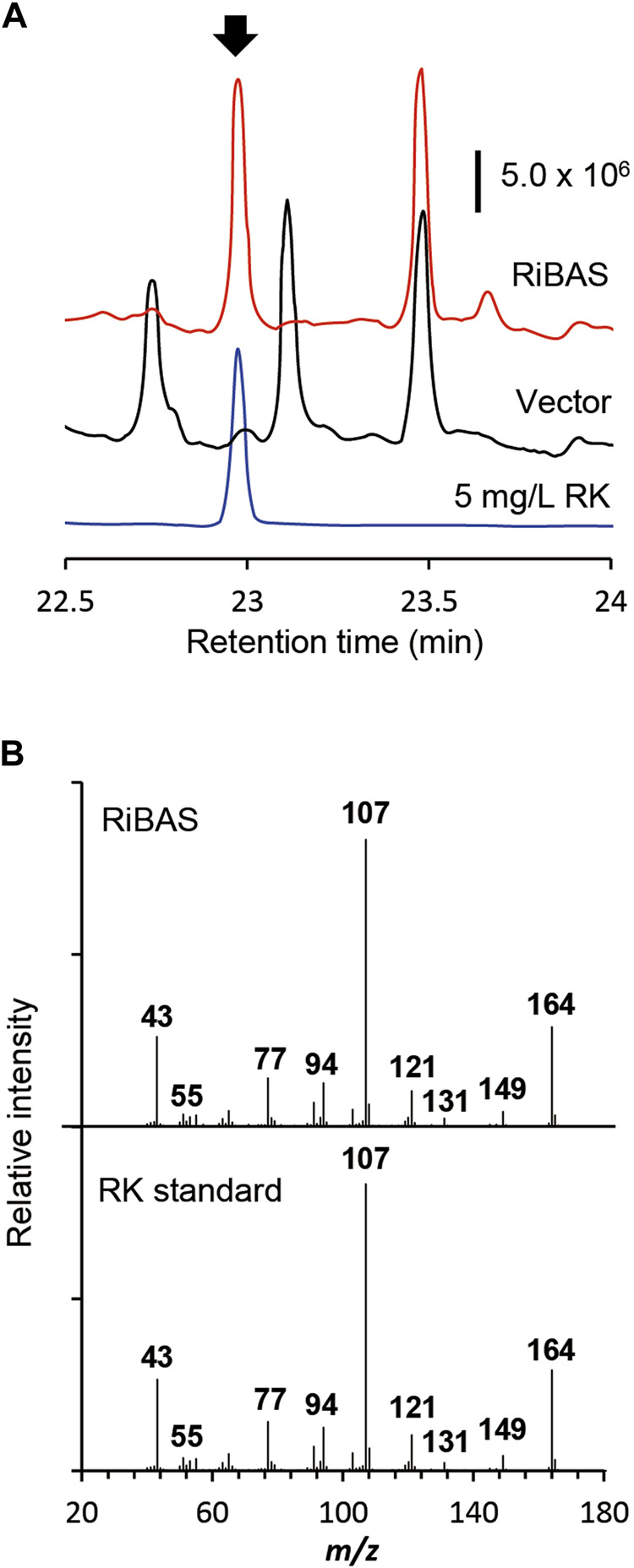
Production of RK from glucose by *E. coli* expressing 4CL and BAS. **(A)** GC-MS profile of extracts of cultured *E. coli* AT2 harboring pCDF-Atu4CL-RiBAS (RiBAS) or pCDFduet-1 (Vector). Arrow, peak of standard RK. **(B)** Gas chromatography-mass spectrometry fragmentation patterns of peaks at 23 min (arrow in **(A)**).

We used AT2 as a heterologous expression host to construct the RK pathway. We expressed both the genes for AtCL and RiBAS, derived from *Agrobacterium tumefaciens* C58 (Campillo et al., 2014) and *Rubus idaeus* BAS (Zheng and Hrazdina, 2008) in the AT2 strain under the control of T7lac promoter. The resulting AT2Ri strain was cultured in modified M9 medium for 169 h and its metabolites were extracted with ethyl acetate and analyzed by GC-MS (Figure 4A). The ion peaks had the same retention time and fragmentation ion pattern as standard RK in the metabolites of AT2Ri, but not AT2 (Figures 4A,B). The AT2Ri strain produced 0.44 mg L^−1^ of RK from glucose for 76 h, whereas is considerable amount of *p*CA remaining in the culture supernatant (Table 3), implied insufficient BAS activities in the cells. Substituting serine 331 with valine in BAS from *Rhemu palmatum* (RpBAS) increases the catalytic activity (Abe et al., 2007). Alignment of the amino acid sequences showed that this serine residue was conserved in RiBAS (Supplementary Figure S3). The corresponding mutants of RiBAS (RiBAS^S338V^), RpBAS, and mutated RpBAS^S331V^, together with AtCL, were introduced into AT2, respectively. The resulting AT2Rp and AT2RpSV strains, expressing RpBAS and RpBAS^S331V^, produced 28 and 29 mg/L of RK from glucose as the raw material after 76 h, respectively. These yields were 60-fold higher than that of AT2Ri (Table 3). The AT2Rp and AT2RpSV strains accumulated less *p*CA than AT2Ri, indicating that conversion of *p*CA to RK is more efficient with RpBAS and RpBAS^S331V^. Our study showed that the mutations in RpBAS and RiBAS had little effect on cellular RK production (Table 3), probably due to the pH dependence of BAS activity (Abe et al., 2007). Less *p*-hydroxybenzalacetone accumulated in cultured AT2RpSV (Table 3), implying sufficient BAR activities in this strain under appropriate culture conditions.

**TABLE 3:** Raspberry ketone production by *E. coli* expressing different BAS genes.

Gene	Tyrosine (mg/l)	*p*-Coumaric acid (mg/l)	*p*-Hydroxybenzalacetone (mg/l)	Raspberry ketone (mg/l)
RiBAS	31.3 ± 1.9	142.6 ± 8.3	0.04 ± 0.01	0.44 ± 0.02
RpBAS	6.4 ± 0.5	<0.1	3.44 ± 0.32	28.38 ± 3.52
RiBAS (S338V)	27.6 ± 1.8	353.4 ± 17.6	0.07 ± 0.01	0.29 ± 0.02
RpBAS (S331V)	<0.1	0.6 ± 0.1	0.46 ± 0.08	29.19 ± 2.32

AT2 harboring BAS expression plasmids were cultured in modified fermentation medium containing 1% glucose for 76 h, then concentrations of compounds were determined by LC-MS.

### 3.3 Genetic and Chemical Manipulation Increased Intracellular Malonyl-CoA and RK Production.

**FIGURE 5:**
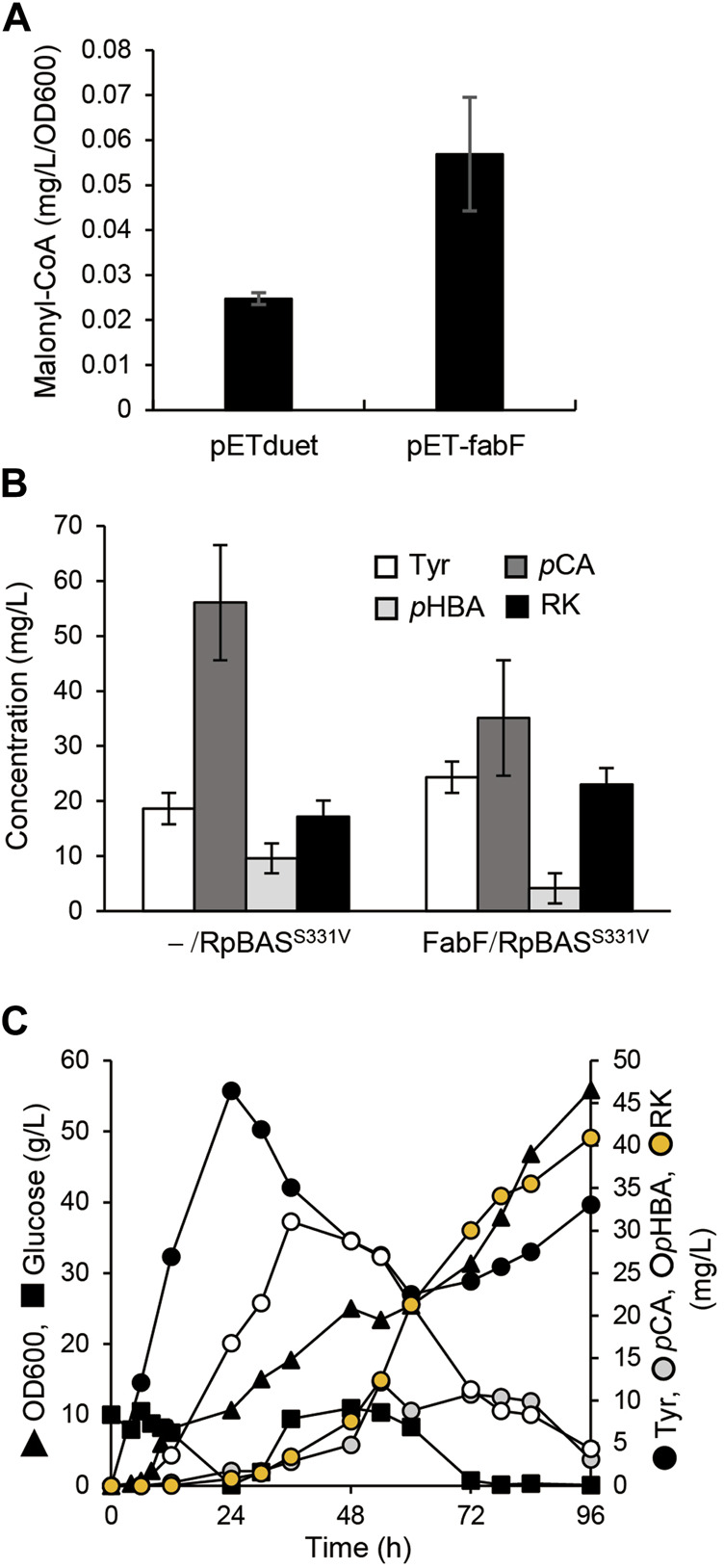
Overexpression of FabF increased intracellular malonyl-CoA and RK production. **(A)** Intracellular malonyl-CoA concentration in *ΔtyrR* strain harboring pETduet-1 or pET-fabF incubated in modified fermentation medium at 30°C for 60 h. **(B)** Concentrations of RK and intermediates in cultured AT2 strain harboring pCDF-Atu4CL-RpBAS^S331V^ (-/RpBAS^S331V^) or pCDF-Atu4CL-RpBAS^S331V^-fabF (FabF/RpBAS^S331V^ (AT3RpSV strain). Strains were incubated in modified fermentation medium at 30°C for 60 h. **(C)** Fed-batch cultures of AT3RpSV in jar fermenters containing 0.5 L of fermentation medium were incubated for 24 h at 30°C and pH 7.1 (maintained by 10% ammonium), then fed with glucose (0.8 g/L/h). Error bars indicate standard deviation (*n* = 3).

The overexpression of *fabF*, which encodes β-ketoacyl carrier protein synthase II, inhibits FabD activity and hence fatty acid elongation (Figure 1), and resulted in cellular accumulation of malonyl-CoA in *E. coli* (Subrahmanyam and Cronan 1998; Kassab et al., 2019). The AT3 strain that was the AT2 strain overexpressing in under the control of T7 lac promoter, accumulated 2.2-fold more malonyl-CoA than AT2 (Figure 5A). We constructed a plasmid to overexpress *fabF* along with the AtCL and RpBAS^S331V^ genes and introduced it into AT2 to generate AT3RpSV, which produced 24 mg/L of RK when cultured for 60 h in modified fermentation medium (Figure 5B). The production titer of AT3RpSV was 1.4-fold higher than that of AT2RpSV, indicating that the increased cellular malonyl-CoA levels improved the amount of malonyl-CoA available for RK production. We optimized aeration conditions, culture media, and IPTG concentrations for RK production by AT3RpSV (Supplementary Figure S4). Fed-batch cultured AT3RpSV under controlled glucose addition produced 41 mg/L of RK with a production yield of 0.08% (vs glucose, Figure 5C).

**FIGURE 6:**
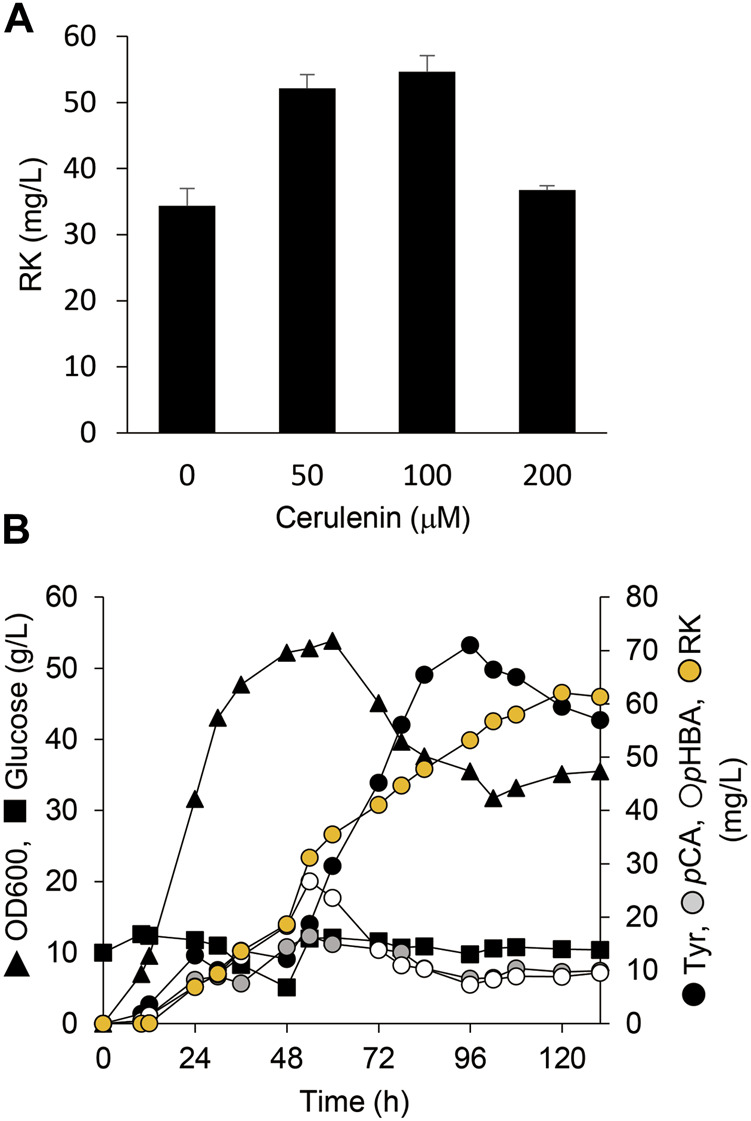
Cerulenin addition increased *E. coli* RK production. **(A)** Production of RK by AT3RpSV cultured in modified fermentation medium containing various concentrations of cerulenin at 30°C for 60 h. **(B)** Fed-batch cultures of AT3RpSV were incubated in jar fermenters containing 0.5 L of fermentation medium at 30°C and pH 7.1 (maintained by 10% ammonium). Cerulenin (100 mM) was added after incubation for 10 h and the cultures were fed with glucose (0.8 g/L/h) after incubation for 24 h. Error bars indicate standard deviation (*n* = 3).

Cerulenin binds to ACP synthase to block its interaction with malonyl-CoA and inhibits fatty acid biosynthesis. Adding cerulenin to bacterial cultures thus accumulates high levels of intracellular malonyl-CoA (Davis et al., 2000). We simultaneously added IPTG and various concentrations of cerulenin to AT3RpSV cultures. The AT3RpSV strain produced 1.6-fold RK (54 mg/L) when cultured with 0.1 mM cerulenin than those without cerulenin (Figure 6A). Fed-batch cultures of AT3RpSV with 0.1 mM cerulenin in 1-L jar fermenters produced 62 mg/L of RK under optimized culture conditions, which increased the yield (Figure 6B) up to 0.12% vs glucose.

## 4 Discussion

We constructed a microbial platform that produced RK from glucose. Genetically engineering *E. coli* metabolism and subsequent PAL optimization resulted in the respective production of 19.3 and 1.9 g/L of Tyr and *p*CA from glucose (Figures 2, 3). A strain producing *p*CA harbored plasmids to express CL and BAS generated RK from glucose (Figure 4). Increasing cellular malonyl-CoA by genetic and chemical manipulation improved the RK yield (Figures 5, 6). Finally, fed-batch culture under optimal conditions fermented 62 mg/L of RK from glucose (Figure 6). Currently, the use of cerulenin is a disadvantage for cost-saving RK production. Notably, our platform produces 41 mg/L of RK without the cerulenin, which is 12-times more than the previous fermenting process (Lee et al., 2016).

During biosynthesis, BAS conjugates *p*-coumaroyl-CoA and malonyl-CoA, which are respectively synthesized *via* the phenylpropanoid and fatty acid synthesis pathways (Figure 1). The efficient production of RK from glucose in a heterologous host requires the optimal production of these substrates. Due to difficulties achieving this, published reports describing microbial RK production are limited to those in which phenylpropanoid *p*CA was the raw material (Lee at al. 2016; Wang et al., 2019; Milke et al., 2020). The present study engineered the phenylpropanoid- and fatty acid synthesis pathways using genetic and chemical approaches, as well as fermentation, and optimized *p*CA levels and malonyl-CoA supplies in *E. coli*.

The engineered AT2 strain fermented glucose to produce 1.9 g/L of *p*CA during culture for 60 h (Figure 3). This amount exceeded that produced by any previous *E. coli* system (Flourat et al., 2021). Not only is *p*CA important as an aromatic precursor of bioactive substances such as stilbenoids, flavonoids and curcuminoids (Katsuyama et al., 2008; Trantas et al., 2009), but it is also a raw material for thermo-tolerant plastics (Kaneko et al., 2006). Our platform could thus facilitate the production of these valuable compounds.

The culture supernatant of the AT2 strain still contained a large amount of tyrosine (>1.0 g/L), and this strain yielded 10-fold less *p*CA than AT1 (Figures 2, 3). This implied insufficient conversion of tyrosine to *p*CA, and that enhancing RgPAL production improves the generation of *p*CA and RK. The tyrosine conversion rate of RgPAL to *p*CA reached 0.8 g of *p*CA/L/h under resting-cell reactions (Table 2). This was much higher than the calculated maximum rate of 0.07 g of *p*CA/L/h generated by fermenting AT2 cells (Figure 3). This indicates that fine-tuning cellular metabolic flux further improves productivity.

The availability of intracellular malonyl-CoA was increased by FabF overexpression and cerulenin, which improved RK production *de novo* (Figures 5, 6). This implied that increasing the intracellular flux for malonyl-CoA synthesis would improve RK production. Another strategy might be to overproduce acetyl-CoA carboxylase that converts intracellular acetyl-CoA to malonyl-CoA (Figure 1), which is supported by the following findings. Excessive acetyl-CoA carboxylase production results in a 100-fold increase in intracellular malonyl-CoA levels (Davis et al., 2000). Overexpressed acetyl-CoA carboxylase combined with engineering the metabolism of the glycolytic system and the TCA cycle, results in 4- and 5.6-fold increases, respectively, in the amounts of intracellular malonyl-CoA and naringenin, a plant-specific secondary metabolite derived from malonyl-CoA (Xu et al., 2011).

This study used multiple plasmids for expressing heterologous genes. Although this approach allows rapid construction of recombinant *E. coli*, several issues should be addressed before applied to large-scale fermentation that includes stability of plasmids, cost of antibiotics, and stable enzyme production. Genomic integration of the introduced genes should be a possible approach to solve these issues. CRISPR/Cas9 or λ-Red recombination systems enable scarless genome engineering in *E. coli* (Pyne et al., 2015; Bayer et al., 2021; Vo et al., 2021) and must be powerful tools for this purpose. These approaches improve our RK fermentation system to a more stable and economically friendly one.

In conclusion, this study established a microbial system that produced useful amounts of RK at low cost, and within a reasonable time frame. The present supply of RK derived from plants is limited and extraction is costly. Our straightforward batch fermentation system offers an inexpensive and efficient alternative to extracting RK from plants based on a simple carbon source that should significantly contribute to the flavor and fragrance industries.

## Data Availability

The datasets presented in this study can be found in online repositories. The names of the repository/repositories and accession number(s) can be found in the article/Supplementary Material.
