# Supplementary material for: Glucose-Derived Raspberry Ketone Produced via Engineered Escherichia coli Metabolism
Source: Front Bioeng Biotechnol. 2022 Feb 14;10:843843. doi: 10.3389/fbioe.2022.843843 (PMC8883332; doi:10.3389/fbioe.2022.843843)
Supplement: Supplementary file 1 [file DataSheet1.PDF]

Glucose-derived raspberry ketone produced *via* engineered *Escherichia coli* metabolism

Shunsuke Masuo, Chisa Saga, Kurumi Usui, Yuma Sasakura, Yukie Kawasaki, and Naoki Takaya*

Faculty of Life and Environmental Sciences, Microbiology Research Center for Sustainability, University of Tsukuba, Tsukuba, Ibaraki 305-8572, Japan

Corresponding Author: Naoki Takaya

E-mail: takaya.naoki.ge@u.tsukuba.ac.jp

Tel.: 81-29-853-4937

Fax:81-29-853-7191

Table S1. Strains used in this study.

E. coli strains	Relevant genotype	Reference
JM109	*endA1 recA1 gyrA96 thi-1 hsdR17* (r_K^-, m_K^+) *relA1 supE44* Δ(lac-proAB) / F' [*traD36 proA*$^+$*B*$^+$ *lacI*^{q} *lacZ*Δ*M15*]	Novagen
BL21(DE3)	F^- *ompT hsdS*$_B$(r_B^- m_B^-) *gal dcm* λ(DE3)	Novagen
NST37(DE3) /ΔpheLA	*aroG39 aroF394 PheA101 pheO352 tyrR366 tyrA4 trpE401 lacY5 malT384 thi-1* λ(DE3) *ΔpheLA*	Masuo et al. 2016
AT	BL21(DE3) harboring pET-tyrA/pACYC-aroG4	This study
NT	NST37(DE3)/ΔpheLA harboring pET-tyrA/pACYC-aroG4	This study
PO	BL21 (DE3) *ΔpoxB*::Km^r	This study
TY	BL21 (DE3) *ΔtyrR*::Zeo^r	This study
AT1	TY harboring pET-tyrA/pACYC-aroG4	This study
AT2	TY harboring pET-tyrA/pACYC-aroG4/pRSF-Rgpal	This study
AT2Ri	TY harboring pET-tyrA/pACYC-aroG4/pRSF-Rgpal/pCDF-AtCL-RiBAS	This study
AT2RiSV	TY harboring pET-tyrA/pACYC-aroG4/pRSF-Rgpal/pCDF-AtCL-RiBAS^{S338V}	This study
AT2Rp	TY harboring pET-tyrA/pACYC-aroG4/pRSF-Rgpal/pCDF-AtCL-RpBAS	This study
AT2RpSV	TY harboring pET-tyrA/pACYC-aroG4/pRSF-Rgpal/pCDF-AtCL-RpBAS^{S331V}	This study
AT3	TY haroring pET-fabF	This study
AT3RpSV	TY harboring pET-tyrA/pACYC-aroG4/pRSF-Rgpal/pCDF-AtCL-RpBAS^{S331V}-fabF	This study
BFev	BL21 (DE3) harboring pET-FevV	This study
BRg	BL21 (DE3) harboring pET-28a-pal	This study
BCs	BL21 (DE3) harboring pET-Cspal	This study
BLe	BL21 (DE3) harboring pET-Lepal	This study

Table S2. Plasmids used in this study.

Plasmid	Description	Reference
pETduet-1	Expression vector. Ampicillin resistance (Amp^r).	Novagen
pCDFduet-1	Expression vector. Streptomycin resistance (Sm^r).	Novagen
pRSFduet-1	Expression vector. Kanamycin resistance (Km^r).	Novagen
pACYC-aroG4	$aroG^{fbr}$ under the control of native *aroG* promoter. Chloramphenicol resistance (Cm^r).	Masuo et al. 2016
pET-FevV	*fevV* (TAL gene from *Streptomyces* sp. WK-5344) under the control of T7 promoter (Amp^r).	Kawaguchi et al. 2017
pET-28a-pal	*Rgpal* (PAL gene from *Rhodotorula glutinis*) under the control of T7 promoter (Amp^r).	Zhu et al. 2013
pET-Cspal	*Cspal* (PAL gene from *Camellia sinensis*) under the control of T7 promoter (Amp^r).	This study
pET-Lepal	*Lepal* (PAL gene from *Lithospermum erythrorhizon*) under the control of T7 promoter (Amp^r).	This study
pET-tyrA	*tyrA* under the control of T7 promoter (Amp^r).	This study
pRSF-Rgpal	*Rgpal* under the control of T7 promoter (Km^r).	This study
pCDF-AtCL-RiBAS	*AtCL* (CL gene from *Agrobacterium tumefaciens*) and *RiBAS* (BAS gene from *Rubus idaeus*) under the control of T7 promoters (Sm^r).	This study
pCDF-AtCL-RiBAS^{S338V}	*AtCL* and *RiBAS*^{S338V} under the control of T7 promoters (Sm^r).	This study
pCDF-AtCL-RpBAS	*AtCL* and *RpBAS* (BAS gene from *Rhemu palmatum*) under the control of T7 promoters (Sm^r).	This study
pCDF-AtCL-RpBAS^{S331V}	*AtCL* and *RpBAS*^{S331V} under the control of T7 promoters (Sm^r).	This study
pET-fabF	*fabF* under the control of T7 promoter (Amp^r).	This study
pCDF-AtCL-RpBAS^{S331V}-fabF	*AtCL, RpBAS*^{S331V} and *fabF* under the control of T7 promoters (Sm^r).	This study

Table S2. List of primers.

Name	Nucleotide sequence (5'-3')	Used to generate
tyrA_fw	ACTTTAAGAAGGAGATATACCATGGTTGCTGAATTGACC	*E. coli tyrA*
tyrA_rv	CAGGCGCGCCGAGCTCGAATTCGGATCCTTACTGGCGATTGTCATTC	
DpoxB_fw	TCAGATGAACTAAACTTGTTACCGTTATCACATTCAGGAGATGGAGAACCAATTAACCCTCACTAAAGGGCG	*poxB* disruption
DpoxB_rv	CGTAAATCAATCATGGCATGTCCTTATTATGACGGGAAATGCCACCCTTTTAATACGACTCACTATAGGGCTC	
C1	GGCTATTTAACCGTTAGTGC	Diagnostic PCR for *poxB* disruption
C2	CCATCATCGCTTCGAGCATG	
DtyrR_fw	ATAGTGTCATATCATCATATTAATTGTTCTTTTTTCAGGTGAAGGTTCCCAATTAACCCTCACTAAAGGGCG	*tyrR* disruption
DtyrR_rv	AGCATAATTTAATATGCCTGATGGTGTTGCACCATCAGGCATATTCGCGCTAATACGACTCACTATAGGGCTC	
C3	TGACAGAAACCTTCCTGCTATC	Diagnostic PCR for *tyrR* disruption
C4	ATTACGAAGCAGCTCTGGCTGTAC	
AtCL_fw	CCGAATTCGATGAGTGTGAAACTTTGGTCGCC	*A. tumefaciens* Atu1416
AtCL_rv	CGGTCGACTCATGCTGCTACCTCTCTGCC	
RiBASmuFw	GAGTATGGGAACATGGTGTCTGCGTGTGTGTTG	RiBAS^{S338V}
RiBASmuRv	CAACACACACGCAGACACCATGTTCCCATACTC	
RpBASmuFw	GACTATGGGAACATGTCAAGTGCGACCGTGTTC	RpBAS^{S331V}
RpBASmuRv	GAACACGGTCGCACTTGACATGTTCCCATAGTC	
fabF_fwCDF	ACTTTAATAAGGAGATATACCATGTCTAAGCGTCGTGTAG	*E. coli fabF* (pCDF-fabF construction)
fabF_rvCDF	TTAAGCATTATGCGGCCGCATTAGATCTTTTTAAAGATCAAAGAAC	
fabF_fwET	ACTTTAAGAAGGAGATATACCATGTCTAAGCGTCGTGTAG	*E. coli fabF* (pET-fabF construction)
fabF_rvET	AGTGGTGGTGGTGGTGGTGCTTAGATCTTTTTAAAGATCAAAGAAC	
pET28b-fabF_fw	GTCTACTAGCGCAGCTTAATGCGAAATTAATACGACTCACTATAGGG	Partial region of pET-fabF (pCDF-AtCL-RpBAS^{S338V}-fabF construction)
pET28b-fabF_rv	TGGCAGCAGCCTAGGTTAATTGGCAGCAGCCAACTCAG	

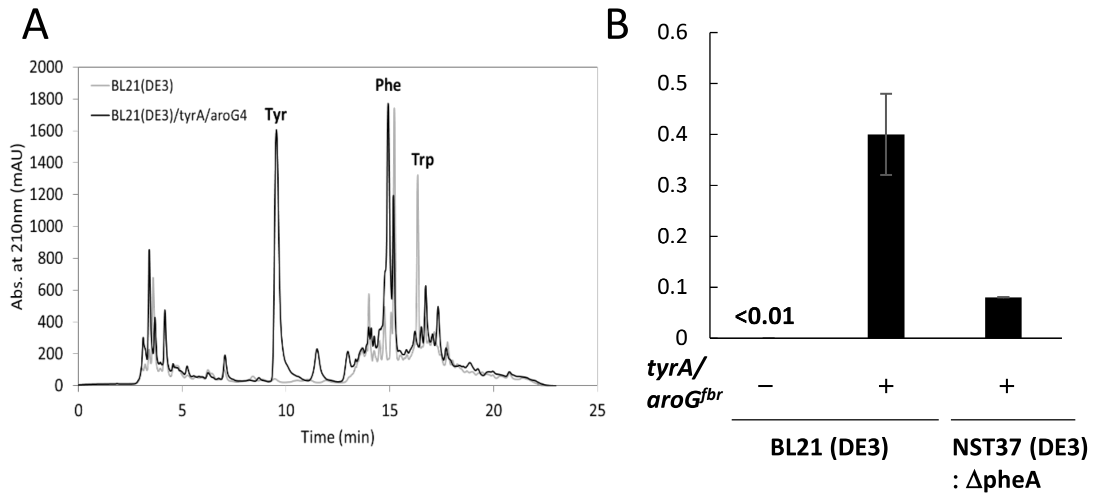

Figure S1. (A) HPLC findings of BL21 (DE3) and BL21 (DE3) harboring pET-tyrA and pACYC-aroG^{fbr}. (B) Tyrosine production by BL21 (DE3) and NST37 (DE3):ΔpheA.
E. coli strains retained either pET-tyrA/pACYC-aroGfbr or empty plasmids, and were cultured in fermentation medium at 30°C for 60 h.

Fig. S2

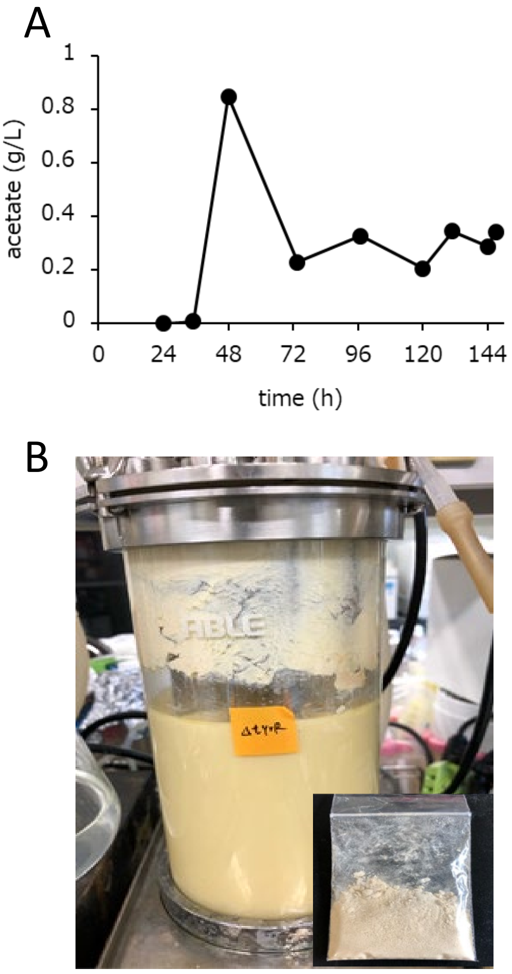

Figure S2. Acetate concentrations and sediments produced by *E. coli* AT1
(A) Acetate concentrations in AT1 fed-batch culture (Fig. 2C). (B) White sediments produced by AT1 were resolved in 0.1 M NaOH and centrifuged to remove debris. Tyrosine was precipitated from the supernatant at pH 7.0 with 0.1 M HCl and dried (Inset).

Fig. S3

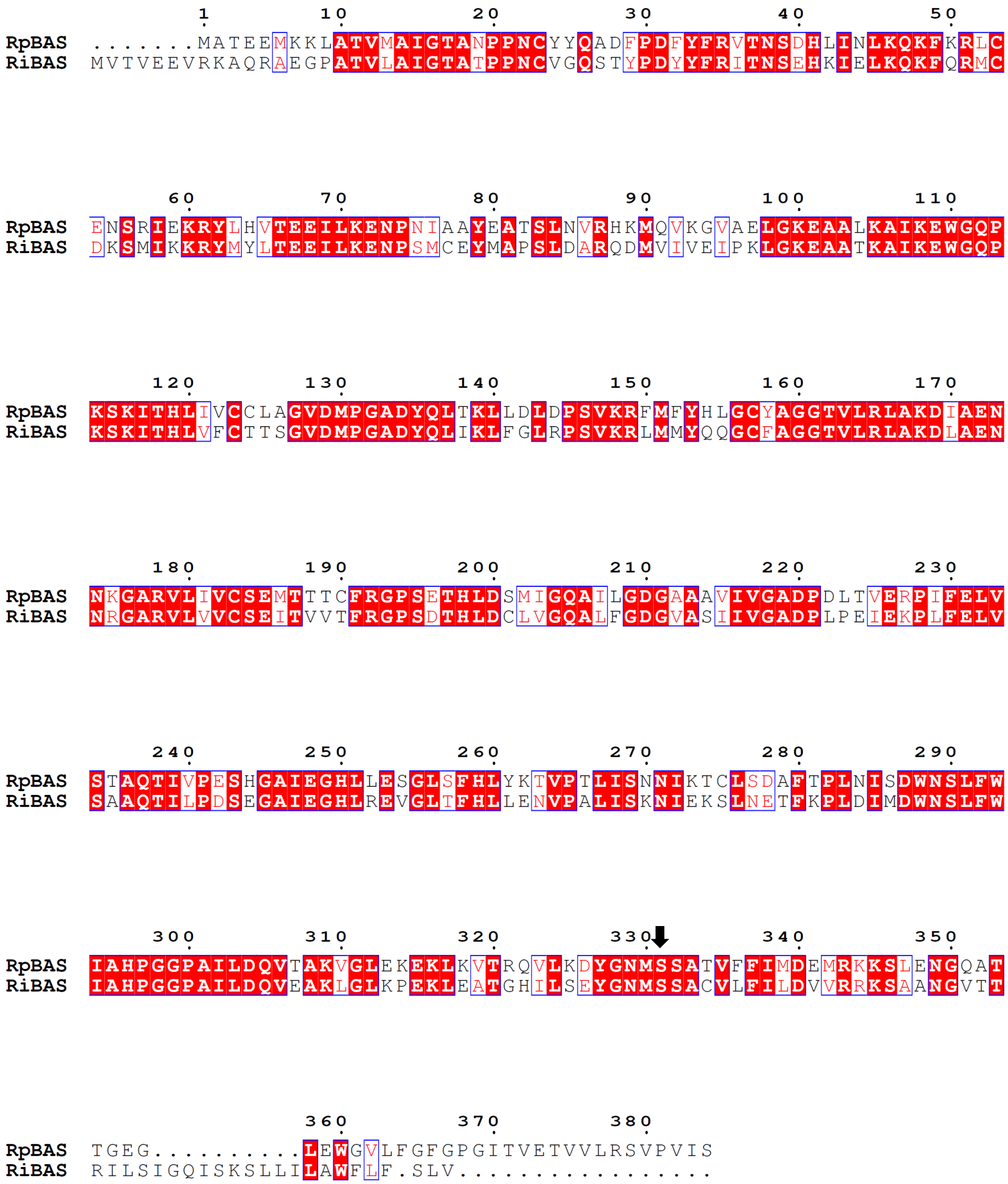

Figure S3. Alignment of amino acid sequences between BAS of *Rubus idaeus* (RiBAS) and *Rhemu palmatum* (RpBAS). Arrow indicates mutated serine residue.

Fig. S4

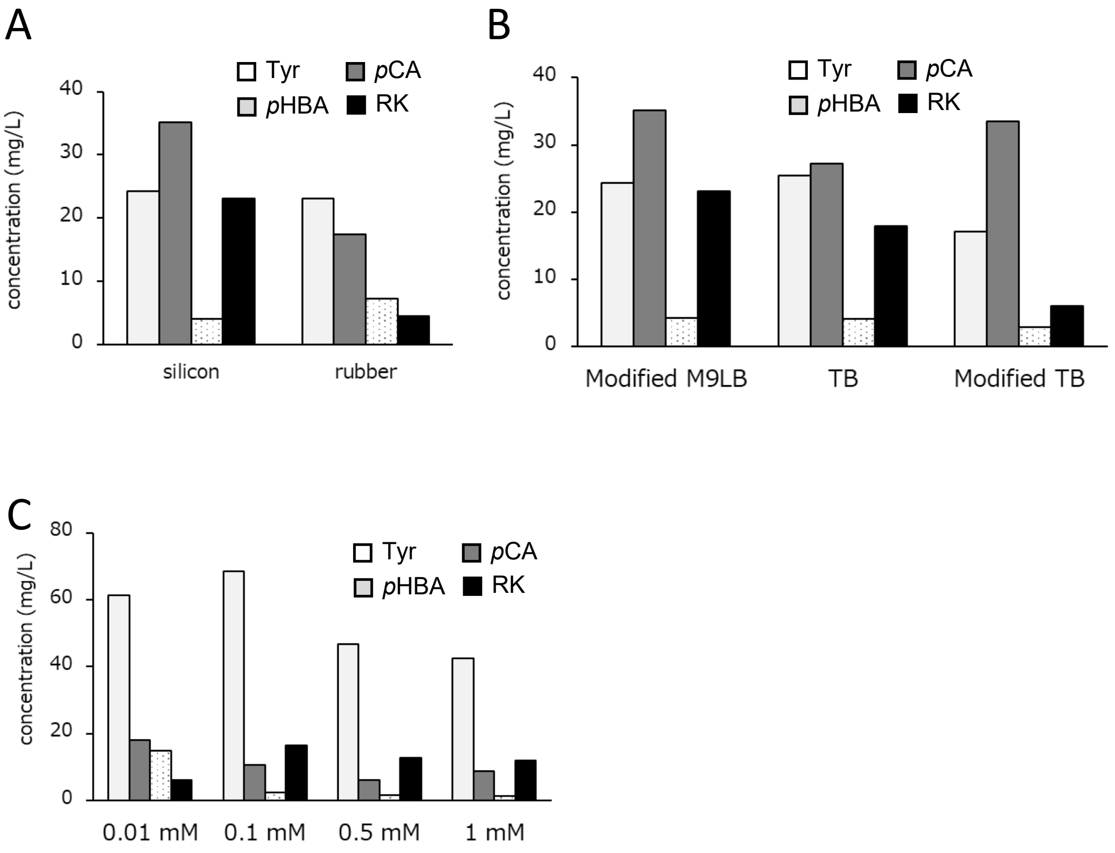

Figure S4. Production of RK by *E. coli* AT3RpSV strain under various conditions.
Each of aeration (A), medium (B), and IPTG concentrations (C) were varied. AT3RpSV was cultured in modified fermentation medium or the indicated medium at 30°C for 60 h. Culture flasks were capped with air-permeable silicon or non-permeable rubber. Modified TB comprised standard TB medium containing 20 mM MOPS (pH 7.0).
